# Epidemiology and treatment patterns of rheumatoid arthritis in a large cohort of Arab patients

**DOI:** 10.1371/journal.pone.0208240

**Published:** 2018-12-19

**Authors:** Soha R. Dargham, Sumeja Zahirovic, Mohammed Hammoudeh, Samar Al Emadi, Basel K. Masri, Hussein Halabi, Humeira Badsha, Imad Uthman, Ziyad R. Mahfoud, Hadil Ashour, Wissam Gad El Haq, Karim Bayoumy, Marianthi Kapiri, Richa Saxena, Robert M. Plenge, Layla Kazkaz, Thurayya Arayssi

**Affiliations:** 1 Weill Cornell Medicine-Qatar, Education City, Doha, Qatar; 2 Hamad Medical Corporation, Doha, Qatar; 3 Jordan Hospital, Amman, Jordan; 4 King Faisal Specialist Hospital and Research Center, Jeddah, Saudi Arabia; 5 Dr. Humeira Badsha Medical Center, Dubai, United Arab Emirates; 6 American University of Beirut, Beirut, Lebanon; 7 Massachusetts General Hospital, Boston, Massachusetts, United States of America; 8 Broad Institute, Cambridge, Massachusetts, United States of America; 9 Merck Research Laboratories, Boston, Massachusetts, United States of America; 10 Tishreen University, Latakia, Syria; Campus Bio-Medico University of Roma, ITALY

## Abstract

**Objectives:**

There is limited information on the epidemiology and treatment patterns of rheumatoid arthritis (RA) across the Arab region. We aim in this study to describe the demographic characteristics, clinical profile, and treatment patterns of patients of Arab ancestry with RA.

**Methods:**

This is a cross sectional study of 895 patients with established rheumatoid arthritis enrolled from five sites (Jordan, Lebanon, Qatar, Kingdom of Saudi Arabia (KSA), and United Arab Emirates). Demographic characteristics, clinical profile, and treatment patterns are compared between the five countries.

**Results:**

The majority of our patients are women, have an average disease duration of 10 years, are married and non-smokers, with completed secondary education. We report a high (>80%) ever-use of methotrexate (MTX) and steroids among our RA population, while the ever-use of disease modifying anti-rheumatic drugs (DMARDs) and TNF-inhibitors average around 67% and 33%, respectively. There are variations in RA treatment use between the five country sites. Highest utilization of steroids is identified in Jordan and KSA (p-value < 0.001), while the highest ever-use of TNF-inhibitors is reported in KSA (p-value < 0.001).

**Conclusion:**

Disparities in usage of RA treatments among Arab patients are noted across the five countries. National gross domestic product (GDP), as well as some other unique features in each country likely affect these. Developing treatment guidelines specific to this region could contribute in delivering standardized therapies to RA patients.

## Introduction

Rheumatoid arthritis (RA) is a chronic autoimmune inflammatory disease associated with progressive joint damage and disability [1]. The prevalence of RA in Northern Europe and North America is estimated at 0.5–1%, and is expected to increase as populations age and mortality decreases [2–4]. In spite of this low prevalence, RA is ranked as the 42^nd^ highest attributable disease to global disability [5], with a two-fold higher morbidity among women compared to men [6]. Other determinants of RA disease include age, socioeconomic status (SES), and ethnicity [7–11].

In the Middle East and North Africa (MENA) region, the epidemiology of RA remains poorly understood with a dearth of data on its prevalence and disease activity among Arab populations. A recent global burden study estimated RA prevalence in MENA region as among the lowest at 0.16% [6]. Based on limited evidence from several MENA regional studies [12–15], RA disease severity and management appear to vary geographically in the region.

Disease modifying anti-rheumatic drugs (DMARDs) are the mainstay treatment for RA prescribed to relieve joint pain and swelling, and to reduce disease activity and disability [16, 17]. Newer biologic agents are typically used in patients with severe unresponsive disease to the classical DMARDs [18, 19]. Although effective, they are significantly expensive, imposing a high economic individual and social burden [20].

In the context of limited data on the epidemiology of RA and its treatment patterns in the MENA region, we aim in the study to describe the demographic, clinical characteristics, and treatment patterns of patients with RA of Arab ancestry living in five Middle Eastern countries.

## Materials and methods

### Study design and sampling

This is a secondary data analysis of a cross-sectional study of RA patients enrolled in the Genetics of Rheumatoid Arthritis in Some Arab States (GRASS) multicenter case-control study [21]. Data were collected between October 2012 and March 2016 from consecutive RA patients attending rheumatology outpatient clinics in Jordan, the Kingdom of Saudi Arabia (KSA), Lebanon, Qatar, and the United Arab Emirates (UAE). To participate in the study, patients had to be older than 18 years of age, of Arab ancestry by self-report, and diagnosed with RA according to the 1987 American College of Rheumatology criteria [22].

All the enrolled patients were adequately informed about the research and provided written consent prior to study enrollment. The study was approved by the ethics boards at all study sites. These include Jordan Hospital Institutional Review Board (Jordan), King Faisal Specialist Hospital and Research Center Institutional Review Board (KSA), American University of Beirut Institutional Review Board (Lebanon), Hamad Medical Center Institutional Review Board and Weill Cornell Medicine-Qatar Institutional Review Board (Qatar), and Dubai Scientific Research Ethics Committee (UAE). A questionnaire was completed by all patients and their physicians capturing sociodemographic and clinical data, including medical history of RA, limited comorbidities and medication use.

### Outcomes and measures

Sociodemographic characteristics collected included self-reported ancestry, nationality, age, sex, education, and marital status. Self-reported ancestry was classified as Gulf, Levant, or Africa if three or more of the patients’ grandparents originated from the Arabian Gulf, Levant, or Africa regions, respectively; all other reported ancestry were grouped in the “other/mix” classification. Education was categorized into three groups: patients who completed primary education or none, middle or secondary school, and college or university education. Marital status was categorized as either never- or ever-married. Data on smoking status, frequency and duration (in years) for both cigarettes and shisha (narghileh) were collected and classified as never- or ever-smoked.

Information on age at diagnosis, duration of disease, presence of rheumatoid factor (RF) and /or anti-cyclic citrullinated peptide (anti-CCP; antibody status), presence of comorbidities (coronary artery disease, peripheral vascular disease, history of stroke, and type II diabetes mellitus (T2DM)), and the use of medications to treat RA were collected from the patients’ medical records. The use of medications was described as never- or ever-use for the following medications: methotrexate (MTX), DMARDs (antimalarial, leflunomide, and sulfasalazine), steroids, and TNF-inhibitors (adalimumab, etanercept, and infliximab) and non-TNF-inhibitors (rituximab) biologics. The use of non-TNF-inhibitor biologics (rituximab) was minimal across all the sites and not included in the analysis. Information on other non-TNFi drugs (tocilizumab and abatacept) were not collected as these medications were not readily available in the countries when the study was initiated in 2012. This impeded us from systematically capturing such data.

### Statistical analysis

Sample characteristics were summarized using frequency distributions except for patients’ age, age at diagnosis, and duration of disease for which the mean and standard deviation were calculated. Socio-demographic, smoking status, co-morbidities, and treatment patterns were compared between the five country sites using the Chi-square and Fisher’s exact tests. One way Analysis of Variance (ANOVA) was used to compare patients’ age, age at diagnosis, and duration of disease between the five country sites. For each of the different types of medication, bivariate and then multivariate logistic regressions were built to assess what factors were associated with their use. Unadjusted and adjusted odds ratios (OR) were reported along with their respective 95% confidence intervals (CI). Since UAE did not collect information on anti-CCP and RF, we conducted a sensitivity analysis excluding the UAE sample to factor anti-CCP and RF in the regression. Significance was defined at the 5% level. All analyses were done using IBM-SPSS version 24.0 (Armonk, NY, USA).

## Results

### Sociodemographic and clinical profile

A total of 895 patients with RA were enrolled from the five participating sites. The majority were female (85%), ever-married (87.5%), and with at least middle or secondary school education (73%). About 42% and 39% reported the Gulf and Levant as their ancestry, respectively. The baseline characteristics are summarized in Table 1.

**Table 1:** Overall demographic and clinical characteristics.

	N	%
**Gender**	** **	** **
Male	135	15.1
Female	760	84.9
**Marital Status**	** **	** **
Single	112	12.5
Ever-married	783	87.5
**Education**	** **	** **
No formal education/Primary	245	27.4
Middle/Secondary School	341	38.1
University	309	34.5
**Cigarette Smoking Status**	** **	** **
Never Smoked	717	80.1
Ever Smoked	178	19.9
**Shisha Smoking Status**	** **	** **
Never Smoked	795	88.8
Ever Smoked	100	11.2
**Self-Reported Ancestry**	** **	** **
Gulf	373	41.7
Levant	347	38.8
Africa	140	15.6
Other	35	3.9
**Nationality**	** **	** **
Expats	285	31.8
Nationals	610	68.2
**Coronary Artery Disease**	** **	** **
No	861	96.3
Yes	33	3.7
**History of Stroke/TIA**	** **	** **
No	880	98.4
Yes	14	1.6
**Peripheral Vascular Disease**	** **	** **
No	874	97.8
Yes	20	2.2
**Diabetes Mellitus—Type II**	** **	** **
No	775	86.7
Yes	119	13.3

Across country sites, significant variability was noted in self-reported ancestry, disease duration, education, marital status, cigarette and shisha smoking, and presence of T2DM (Table 2). In Lebanon, Levant ancestry was most commonly reported by the patients, while Gulf ancestry was predominantly reported by patients in KSA (p-value < 0.001). Patients recruited from the UAE had the shortest mean disease duration as compared to patients recruited from other sites (p-value < 0.001). In terms of education, Qatar had the highest proportion of university educated subjects (significantly different only from UAE), and Lebanon had the highest proportion of middle/secondary school completion, significantly different from Jordan, Qatar and UAE but not KSA (p-value < 0.000). Patients recruited from Lebanon were more likely to be single (p-value < 0.001) and to smoke compared to patients from Jordan, KSA, and Qatar (p-value = 0.004). T2DM was most frequently reported in patients recruited from KSA, followed by patients from Qatar, Jordan, Lebanon and UAE (p-value < 0.001). Lebanon had the highest proportions of recruited patients with untested anti-CCP and RF and accounted for the lowest proportions of subjects with positive anti-CCP (p-value < 0.001) and RF (p-value < 0.001) compared to Jordan, KSA, and Qatar.

**Table 2:** Demographic and clinical characteristics by country site.

	Jordan, N = 206	KSA, N = 149	Lebanon, N = 133	Qatar, N = 266	UAE, N = 141	P-value
	N (%)	N (%)	N (%)	N (%)	N (%)	
**Age**						0.173
Mean; Std. Dev	48.95 (13.95)	50.04 (12.63)	48.79 (14.27)	48.75 (12.19)	46.28 (13.32)	
**Age at Diagnosis**						0.752
Mean; Std. Dev	39.33 (13.37)	38.72 (12.54)	39.22 (13.99)	39.22 (12.23)	40.69 (12.98)	
**Duration of Disease**						<0.001*
Mean; Std. Dev	9.62 (8.46)^e^	11.33 (8.28)^e^	9.59 (9.75)^e^	9.53 (7.99)^e^	5.60 (7.29)	
**Gender**						0.091
Male	34 (16.5)	18 (12.1)	28 (21.1)	41 (15.4)	14 (9.9)	
Female	172 (83.5)	131 (87.9)	105 (78.9)	225 (84.6)	127 (90.1)	
**Marital Status**						0.004*
Single	22 (10.7)^a^^,^^b^	14 (9.4) ^b^	30 (22.6)^a^	28 (10.5) ^b^	18 (12.8) ^b^	
Ever-married	184 (89.3)	135 (90.6)	103 (77.4)	238 (89.5)	123 (87.2)	
**Education**						<0.001*
No formal education/Primary	50 (24.3) ^b^	58 (38.9)^a^	17 (12.8) ^b^	60 (22.6) ^b^	60 (42.6)^a^	
Middle/Secondary School	91 (44.2) ^a^^,^^b^	45 (30.2) ^b^	67 (50.4)^a^	95 (35.7) ^b^	43 (30.5) ^b^	
University	65 (31.6)^a^	46 (30.9) ^a^	49 (36.8) ^a^	111 (41.7) ^a^	38 (27.0)	
**Cigarette Smoking Status**						<0.001*
Never Smoked	151 (73.3)^b^	132 (88.6)^a^	78 (58.6)^c^	226 (85.0) ^a^	130 (92.2) ^a^	
Ever Smoked	55 (26.7)	17 (11.4)	55 (41.4)	40 (15.0)	11 (7.8)	
**Shisha Smoking Status**						<0.001*
Never Smoked	179 (86.9)^a^^,^^b^	138 (92.6)^a^	101 (75.9)^c^	249 (93.6) ^a^	128 (90.8) ^a^	
Ever Smoked	27 (13.1)	11 (7.4)	32 (24.1)	17 (6.4)	13 (9.2)	
**Coronary Artery Disease**						0.092
No	196 (95.1)	140 (94)	129 (97.7)	256 (96.2)	140 (99.3)	
Yes	10 (4.9)	9 (60)	3 (2.3)	10 (3.8)	1 (0.7)	
**History of Stroke/TIA**						0.128
No	201 (97.6)	144 (96.6)	131 (99.2)	263 (98.9)	141 (100)	
Yes	5 (2.4)	5 (3.4)	1 (0.8)	3 (1.1)	0 (0)	
**Peripheral Vascular Disease**						0.173
No	202 (98.1)	147 (98.7)	125 (94.7)	259 (97.4)	141 (100)	
Yes	4 (1.9)	2 (1.3)	7 (5.3)	7 (2.6)	0 (0)	
**Diabetes Mellitus—Type II**						<0.001*
No	182 (88.3)^b^	111 (74.5)^c^	120 (90.9)^a^^,^^b^	225 (84.6)^b^^,^^c^	137 (97.2)^a^	
Yes	24 (11.7)^e^	38 (25.5)	12 (9.1)	41 (15.4)	4 (2.8)	
**Ancestry**						<0.001*
Gulf	43 (20.9)^c^	130 (87.2)^a^	1 (0.8)^d^	123 (46.2)^b^	76 (53.9)^b^	
Levant	138 (67.0)^b^	4 (2.7)^d^	126 (94.7)^a^	44 (16.5)^c^	35 (24.8)^c^	
Africa	24 (11.7)^b^	4 (2.7)^c^	2 (1.5)^c^	83 (31.2)^a^	27 (19.1)^a^^,^^b^	
Other	1 (0.5)^b^	11 (7.4)^a^	4 (3.0)^a^^,^^b^	16 (6.0)^a^	3 (2.1)^a^^,^^b^	
**Nationality**						<0.001*
Expats	82 (39.8)^a^	0 (0.0)	15 (11.3)	133 (50.0)^a^	55 (39.0)^a^	
Nationals	124 (60.2)	149 (100.0)	118 (88.7)	133 (50.0)	86 (61.0)	

*–p-value < 0.05

a,b,c,d,e–groups with similar letter are not statistically different; groups with different letter are statistically different.

### RA treatment profile

**Fig 1:**
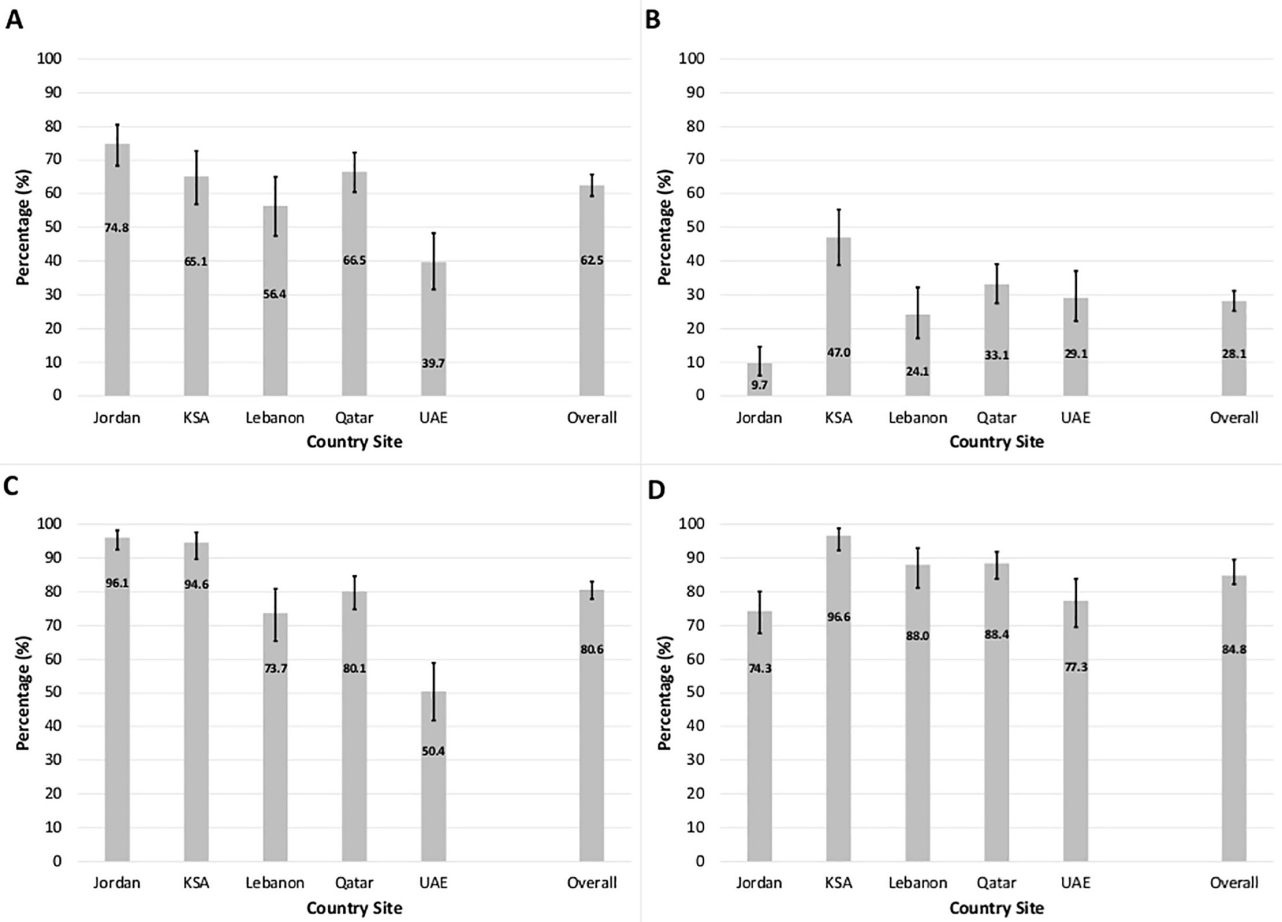
Percentage for ever-use of A) DMARDs, B) TNF-inhibitors, C) steroids, and D) methotrexate among Arab rheumatoid arthritis patients across five country sites.

**Fig 2:**
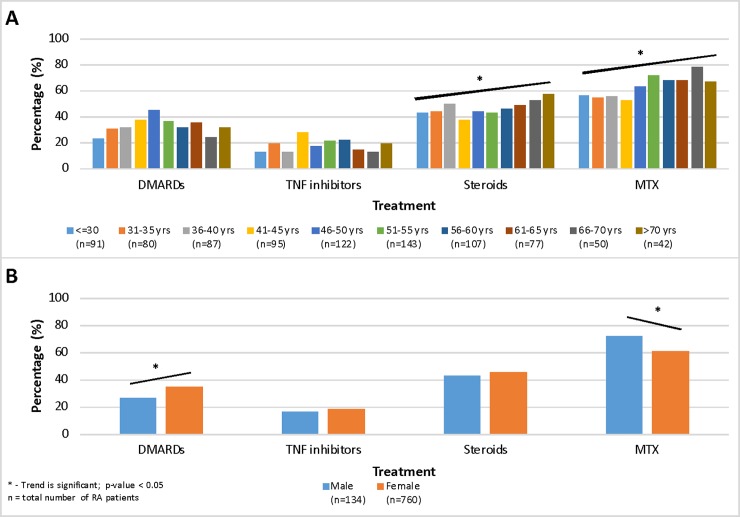
A) Age- and B) Sex-specific trends of treatment utilization among 895 Arab rheumatoid arthritis patients.

Methotrexate (87.8%) and steroids (80.6%) were the most commonly ever-used medications across all sites. Around two thirds of patients ever-used DMARDs other than MTX. Only one third of patients ever- used TNF-inhibitors. Additionally, there were significant differences in the types of medication used between countries (Fig 1). Highest utilization of steroids was identified in Jordan and KSA (p-value < 0.001; Fig 1C), while highest ever-use of TNF-inhibitors was reported in KSA (p-value < 0.001; Fig 1B). The use of DMARDs was highest among female patients (p-value = 0.004) while MTX was more commonly used among male patients (p-value = 0.004; Fig 2B) and the use of both MTX and steroids increased with age (p-value < 0.001; Fig 2A). Apart from TNF-inhibitors, the only other biologic analyzed was rituximab. Its ever-use was higher in KSA, Lebanon and Qatar, in comparison to Jordan (p-value < 0.001). Patients with T2DM were twice as likely to have ever-used rituximab in adjusted analysis. Further, RF was also associated with rituximab ever-use. No other predictive factors were associated with rituximab ever-use (results not shown).

Adjusted analysis (Table 3) showed that the use of methotrexate was significantly positively associated with duration of disease, male sex, and living in KSA, Lebanon, and Qatar as compared to Jordan while the use of steroids was negatively associated with university education level and living in Qatar, Lebanon, and the UAE as compared to Jordan. The use of non-biologic DMARDs among RA patients was significantly associated positively with duration of disease and female sex, while negatively with T2DM and living in all four countries compared to Jordan. The use of TNF-inhibitors was significantly positively associated with duration of diagnosis and country site (all four countries compared to Jordan) while negatively associated with ever-married marital status. The sensitivity analysis (excluding the UAE sample) showed that anti-CCP and RF had no effect on medication patterns (results not shown).

**Table 3:** Unadjusted and adjusted odds ratios (95% confidence intervals) of rheumatoid arthritis treatments among Arab patients.

	DMARDs		TNF-inhibitors		Methotrexate		Steroids	
	OR (95% CI)	aOR (95% CI)	OR (95% CI)	aOR (95% CI)	OR (95% CI)	aOR (95% CI)	OR (95% CI)	aOR (95% CI)
**Age**	0.99 (0.98–1.00)	-	1.00 (0.99–1.01)	-	1.03 (1.01–1.04)*	-	1.03 (1.01–1.04)*	-
**Age at Diagnosis**	0.97 (0.96–0.98)*	0.99 (0.97–1.00)	0.98 (0.97–0.99)*	-	1.01 (0.99–1.02)	-	1.00 (0.98–1.01)	-
**Duration of Disease**	1.06 (1.04–1.08)*	1.05 (1.02–1.07)*	1.04 (1.03–1.06)*	1.05 (1.03–1.07)*	1.05 (1.03–1.08)*	1.05 (1.02–1.08)*	1.10 (1.07–1.14)*	1.07 (1.04–1.11)*
**Female**	1.71 (1.18–2.48)*	1.62 (1.09–2.40)*	1.17 (0.77–1.78)	-	0.36 (0.18–0.73)*	0.33 (0.15–0.70)*	1.17 (0.75–1.83)	-
**Ever Married**	1.16 (0.77–1.74)	-	0.66 (0.43–1.00)*	0.58 (0.37–0.93)*	0.86 (0.48–1.52)	-	2.12 (1.37–3.29)*	1.64 (0.97–2.76)
**Education (vs. Non-schooling)**								
Middle/secondary	1.49 (1.07–2.09)*	1.35 (0.92–1.99)	0.67 (0.46–0.97)*	0.88 (0.58–1.34)	1.26 (0.80–1.98)	-	0.87 (0.56–1.35)	0.75 (0.44–1.26)
University	1.58 (1.12–2.24)*	1.45 (0.96–2.18)	1.12 (0.78–1.61)	1.41 (0.93–2.14)	1.18 (0.75–1.87)	-	0.61 (0.40–0.94)*	0.03 (0.33–0.95)*
**Cigarette Smoking**	0.86 (0.61–1.20)	-	0.64 (0.43–0.95)*	0.81 (0.52–1.25)	1.43 (0.87–2.35)	-	1.58 (1.00–2.51)*	1.39 (0.82–2.36)
**Coronary Artery Disease**	0.81 (0.40–1.63)	-	1.12 (0.52–2.38)	-	2.86 (0.68–12.08)	-	2.45 (0.74–8.11)	-
**Stroke**	0.59 (0.21–1.71)	-	0.42 (0.09–1.90_	-	0.65 (0.18–2.37)	-	3.14 (0.41–24.17)	-
**Type II Diabetes**	0.69 (0.46–1.01)	0.64 (0.41–0.99)*	1.41 (0.94–2.12)*	1.19 (0.75–1.89)	1.54 (0.84–2.82)	-	1.90 (1.06–3.41)*	0.85 (0.43–1.66)
**Country Site (Vs. Jordan)**								
KSA	0.63 (0.40–1.00)	0.61 (0.38–0.99)*	8.24 (4.70–14.46)*	7.73 (4.33–13.80)*	9.98 (3.88–25.66)*	10.12 (3.88–26.41)*	0.71 (0.26–1.94)*	0.65 (0.23–1.79)
Lebanon	0.44 (0.28–0.71)*	0.41 (0.25–0.67)*	3.04 (1.65–5.59)*	2.95 (1.57–5.53)*	2.53 (1.38–4.66)*	2.75 (1.45–5.22)*	0.12 (0.05–0.26)*	0.12 (0.05–0.28)*
Qatar	0.67 (0.45–1.01)	0.65 (0.43–0.99)*	4.60 (2.71–7.79)*	4.51 (2.63–7.73)*	2.63 (1.61–4.28)*	2.79 (1.69–4.62)*	0.16 (0.08–0.35)*	0.16 (0.08–0.36)*
UAE	0.22 (0.14–0.35)*	0.24 (0.15–0.40)*	3.81 (2.12–6.86)*	4.73 (2.56–8.77)*	1.18 (0.71–1.95)	1.57 (0.92–2.67)	0.04 (0.02–0.90)*	0.05 0.02–0.10)*

*–p-value < 0.05

OR: odds ratio; aOR: adjusted odds ratio; CI: confidence interval

## Discussion

Our cross-sectional study examined socio-demographic, clinical, and treatment profile of 895 RA patients of Arab ancestry recruited from five countries in the Middle East. The majority are women, have an average disease duration of 10 years, are married and non-smokers, with completed secondary education, and comorbidities consistent with what has been described in other populations in the world [23, 24]. Our population, however, and unlike patients reported in studies from North America and Europe, are about 10 years younger at the time of diagnosis. A potential explanation could be related to the lower average age of the population in the Middle Eastern countries [25]. However, genetic and environmental factors cannot be excluded. In the Middle East, consanguinity is high and can reach up to 50% in some countries [26], raising the question of genetic anticipation as a potential explanatory factors [27]. Other chronic diseases in the MENA region like breast cancer have a peak age that is about 2 decades younger than the peak age in the Western hemisphere [28, 29].

We observed a high ever-use of MTX and steroids among our patients with little variation across all centers, while the ever-use of DMARDs (other than MTX) and TNF-inhibitors significantly varied. This high prescription of MTX is a substantial improvement from prior reports [12] from the region reflecting acceptance and implementation of global guidelines [13, 30–32].

The significant variations in the use of TNF-inhibitors amongst the sites is likely to be explained by the differences in the healthcare systems as well as patient factors. Total health expenditure per capita ranges from as low as USD 257 in Jordan to as high as USD 2,030 in Qatar [33] and access to TNF-inhibitors is likely to be affected not only by health expenditure but also by the national gross domestic product (GDP) per capita (ranging from USD 9,048 in Jordan to USD 127,481 in Qatar [34]) and the health insurance system. In KSA, Qatar, and UAE, all healthcare, including medications, is free or highly subsidized for nationals by the government, while expats living in the latter countries and nationals of Lebanon and Jordan have to pay out of pocket and pay high premium for health insurance to cover health care expenses. This was observed in other studies where European countries [20, 24, 35] with lower GDP had lesser use.

We established factors associated with the choice of RA treatment in our cohort. Increasing age of diagnosis is inversely associated with use of MTX in parallel to the pattern reported from the UK [36] and Sweden [37], and potentially explained by comorbidities and risk of toxicity. On the other hand, MTX use was associated with increasing age of the patient, implying that once the patient started MTX earlier in their life, they would remain taking it even later. Female sex is also associated with lower use of MTX, however not surprising as the patients in our study are women in their childbearing age and physicians are less likely to prescribe MTX due to its well-known association with congenital malformation in pregnant women [38–41]. Interestingly, we identified higher educational status to be associated with higher use of DMARDs and lower use of steroids. These associations between socio-economic status and RA disease activity/duration and treatment is supported in the literature: RA patients with lower income had higher disease activity and lower initiations of DMARDs [10, 42]. As no information on socioeconomic status is collected in our study, the level of education could be perceived in our study as a proxy for socioeconomic status.

There are biases inherent to a cross-sectional analysis of a convenience sample. The prevalence of co-morbidities and medication use could be underestimated due to recall bias as the information could be missing from the patients’ medical records. Records were not maintained for eligible patients who refused participation, thus we are unable to evaluate the characteristics of those who refused to participate. In addition, there was a lack of a central lab to test for anti-CCP and RF to ensure the accuracy of those results. The missing data on anti-CCP and RF status precluded a complete clinical assessment of RA in our study population.

Despite these limitations, our study fills major gaps on RA epidemiology and characteristics pertaining to RA patients of Arab ancestry in the Middle East. Also, the novelty of our study lays in the description, investigation, and comparison of medication patterns between five nations of different GDP levels in the Eastern Mediterranean region.

In summary, this is the largest study of RA patients from the Middle East. Notable findings include younger age of onset of the disease and significant variations in treatment patterns amongst countries. Future studies should aim to address disease severity, remission rates as well as patient and physician treatment preferences. Lastly, developing region specific guidelines for RA treatment, could result in delivering equitable care to patients.

## Supporting information

S1 File RA_profile_treatment. (SAV)
