# Correction: Epidemiology and treatment patterns of rheumatoid arthritis in a large cohort of Arab patients

**DOI:** 10.1371/journal.pone.0214258

**Published:** 2019-03-18

**Authors:** Soha R. Dargham, Sumeja Zahirovic, Mohammed Hammoudeh, Samar Al Emadi, Basel K. Masri, Hussein Halabi, Humeira Badsha, Imad Uthman, Ziyad R. Mahfoud, Hadil Ashour, Wissam Gad El Haq, Karim Bayoumy, Marianthi Kapiri, Richa Saxena, Robert M. Plenge, Layla Kazkaz, Thurayya Arayssi

The following information is missing from the Funding statement: Publication was funded by the Qatar National Library.
